# Supplementary material for: The genome sequence of the Common Grey moth, Scoparia ambigualis Treitschke, 1829
Source: Wellcome Open Res. 2025 Apr 23;10:205. [Version 1] doi: 10.12688/wellcomeopenres.23998.1 (PMC12290446; doi:10.12688/wellcomeopenres.23998.1)
Supplement: Peer review history

# Reviewer report: Reviewer response for version 1

**DOI:** 10.21956/wellcomeopenres.26475.r126506

**Published:** 2025-07-24

**Authors:** Shiqi Luo

**Affiliations:** 1 China Agricultural University, Beijing, China

This data note describes the chromosome-level genome assembly of a male individual of the Common Grey moth ( *Scoparia ambigualis*) using Pacific Biosciences long-read sequencing and Hi-C sequencing. The overall assembly quality was high. The paper includes basic information and data storage details, along with a comprehensive description of the software and methods used.

 I only have a few comments:

 The authors mentioned that “The assembly was improved by manual curation”—please provide some specific details.

 Please include more parameter details when describing the software usage.

 Pay attention to grammatical issues in certain sentences, such as “decreased the scaffold count by -20.0%.”

Are sufficient details of methods and materials provided to allow replication by others?

Partly

Is the rationale for creating the dataset(s) clearly described?

Yes

Are the datasets clearly presented in a useable and accessible format?

Yes

Are the protocols appropriate and is the work technically sound?

Yes

Reviewer Expertise:

insect population and evolutionary genomics

I confirm that I have read this submission and believe that I have an appropriate level of expertise to confirm that it is of an acceptable scientific standard.

# Reviewer report: Reviewer response for version 1

**DOI:** 10.21956/wellcomeopenres.26475.r123170

**Published:** 2025-06-06

**Authors:** Martina Dalíková

**Affiliations:** 1 The University of Kansas, Lawrence, Kansas, USA

The authors present the methods and basic metrics for a new male genome assembly of *Scoparia ambigualis* generated as part of the Darwin Tree of Life project. This assembly will be a valuable resource for future research. The results are presented succinctly and are appropriate for a data note. Although methods are generally well described, I believe several important details are missing or require more clarification.

 1) The methods state that only one specimen was used for all sequencing. Two rounds of dissection are mentioned, but it is unclear what tissue(s) were actually dissected. Please specify which body parts were removed during each dissection, and then clearly state what remained and how it fits with the whole body DNA isolation mentioned in the paper.  I would also be interested in knowing the total mass of tissue left after the dissection. In the methods, it is mentioned that 20-50 mg was used for Hi-C, and an unspecified amount of tissue was used for DNA isolation and PacBio library preparation.

 2) When estimating genome size with GenomeScope2, what maximum k-mer coverage cutoff did you use? As the estimated genome size is relatively large with less the 1/2 of the genome predicted to be repetitive, it would be interesting to know if different k values were tested, and clarify how you chose the final estimate. Providing an independent flow cytometry C-value would help validate the assembly size.

 3) The manuscript mentions running purge_dups but does not mention any cutoff values. It would be useful to know the calculated/used thresholds, as the automatic deduplication was followed by a manual one.

 4) The authors note that the assembly was manually curated, but they do not provide enough details about how the misassemblies were identified. Please provide more details about the misjoins, missing joins and haplotypic duplication you identified and resolved manually. Especially because you do not provide karyotypic evidence for the chromosomal number you obtained in your assembly, and Crambidae have variable chromosomal numbers, so there is no precedent to verify the final assembly from this angle.

Are sufficient details of methods and materials provided to allow replication by others?

Partly

Is the rationale for creating the dataset(s) clearly described?

Yes

Are the datasets clearly presented in a useable and accessible format?

Yes

Are the protocols appropriate and is the work technically sound?

Yes

Reviewer Expertise:

cytogenetics, genomics

I confirm that I have read this submission and believe that I have an appropriate level of expertise to confirm that it is of an acceptable scientific standard.
